# Impact of Dental Rehabilitation on Oral Health-Related Quality of Life in Children with Cerebral Palsy: A 12-Month Prospective Pilot Study in Two Different Age Groups

**DOI:** 10.3390/medicina62071387

**Published:** 2026-07-17

**Authors:** Alaz Oya Erenel, Seçil Çalışkan, Coşkun Yarar, Arife Derda Yücel Şen, Sibel Özdemir

**Affiliations:** 1 Department of Pediatric Dentistry, Faculty of Dentistry, Eskişehir Osmangazi University, Eskisehir 26040, Turkey; sclctn@hotmail.com; 2 Department of Pediatric Neurology, Faculty of Medicine, Eskişehir Osmangazi University, Eskisehir 26040, Turkey; cyarar@ogu.edu.tr (C.Y.); derdayucel@hotmail.com (A.D.Y.Ş.); 3 Private Practice, Eskisehir 26040, Turkey; drsibelkaraca@hotmail.com

**Keywords:** cerebral palsy, oral health-related quality of life, dental rehabilitation, ECOHIS, POQL, pediatric dentistry, special health care needs

## Abstract

*Background and Objectives:* Children with cerebral palsy (CP) are at increased risk for oral health problems, yet prospective evidence on the impact of dental rehabilitation on oral health-related quality of life (OHRQoL) in this population remains limited. This study aimed to prospectively evaluate the effects of comprehensive chairside dental rehabilitation on OHRQoL in children with spastic-type CP across two age groups, and to compare outcomes with healthy controls over a 12-month follow-up period. *Materials and Methods:*This prospective controlled pilot study included 22 children (11 with spastic-type CP, 11 healthy controls), individually matched by age, sex, and caries index (dmft/DMFT). Participants were divided into two subgroups based on dentition stage: the primary dentition group (*n* = 12; 6 CP, 6 controls; ages 4–6, assessed with the parent-reported ECOHIS) and the mixed dentition group (*n* = 10; 5 CP, 5 controls; ages 7–10, assessed with the POQL, which incorporates both child-reported and parent-reported forms). OHRQoL was measured at baseline, 3, 6, and 12 months. Comprehensive chairside dental rehabilitation was performed in accordance with AAPD guidelines. The Friedman test and Mann–Whitney U test were used for within-group and between-group comparisons, respectively. Effect sizes (Kendall’s W and r) and 95% confidence intervals were calculated for statistically significant findings. *Results:* Parent-reported POQL scores demonstrated statistically significant improvement over 12 months in both the CP (*p* = 0.004) and control (*p* = 0.007) groups, with the most pronounced change occurring between baseline and the 12th month. ECOHIS scores and child-reported POQL scores decreased across time points without reaching statistical significance. No significant between-group differences were detected at any time point. *Conclusions:* Comprehensive chairside dental rehabilitation was associated with significant improvements in parent-reported OHRQoL in both children with CP and healthy controls, whereas ECOHIS and child-reported POQL scores showed numerical improvements that did not reach significance. These findings indicate that high-functioning children with spastic-type CP can benefit from dental rehabilitation comparably to their healthy peers, though larger and more heterogeneous samples are needed to confirm and generalize these preliminary results.

## 1. Introduction

Cerebral palsy (CP) is a heterogeneous neurodevelopmental disorder characterized by permanent impairments in motor functions due to brain damage occurring in the prenatal, perinatal, or postnatal periods [1]. In children with CP, motor control impairments, limitations in oral motor functions, and difficulties in swallowing and chewing can hinder the maintenance of oral hygiene, thereby increasing the risk of dental caries, periodontal disease, and oral trauma [2,3]. Furthermore, clinical features associated with CP—such as spasticity, malocclusion, and impaired drooling control—can lead to both functional and aesthetic problems, affecting the child’s social interactions and daily activities [4,5]. Compared to their typically developing peers, children with CP consistently demonstrate poorer oral health-related quality of life (OHRQoL), characterized by more frequent oral pain, difficulties with eating and speaking, and greater emotional and social impact differences that have been attributed primarily to motor limitations impairing oral hygiene, higher caries and periodontal disease burden, and the increased dependency on caregivers for oral care [6,7,8].

Providing oral care for a child with CP requires additional time, training, and resources for caregiving families, which may increase the caregiver burden and psychosocial stress levels, affecting both the quality of care and the family’s quality of life [4]. Since the deterioration of oral health indicators in the CP population is associated with symptoms such as pain, nutritional difficulties, and speech problems, reporting only clinical findings may not fully reflect the experiences of patients and families [5]. Therefore, OHRQoL measurements are critical tools for assessing pain, functional limitations, emotional and social impacts, and the burden on the family beyond clinical parameters [6].

While there are studies in the existing literature examining the OHRQoL levels of children with CP, it has been reported that most of these are cross-sectional in design, and studies prospectively monitoring changes over time following treatment are limited [9,10]. Although the positive effects of dental rehabilitation on pain, function, and care requirements in children with CP have been reported, the evidence evaluating the short- and long-term effects of these interventions on OHRQoL through comparative and repeated measures remains insufficient [11]. Furthermore, since the clinical heterogeneity of CP and age-related developmental differences may influence OHRQoL responses, these variables need to be thoroughly defined in studies and controlled within analyses [3,8]. In this context, the prospective monitoring of children with CP alongside healthy controls is of significant importance for assessing the effects of dental rehabilitation on OHRQoL at baseline, short-term, and mid-term, as well as for revealing the progression of both clinical and family-centered outcomes over time [9]. Specifically, few prospective studies have systematically evaluated whether children with CP derive the same magnitude of OHRQoL benefit from dental rehabilitation as healthy peers, or whether developmental stage modifies this response—leaving a substantial research gap regarding the longitudinal trajectory of OHRQoL in this population [8,9,10].

The aim of this study is to prospectively evaluate the effects of comprehensive chairside dental rehabilitation on the OHRQoL in children with spastic-type CP across two developmental age groups, in comparison with healthy peers. The null hypothesis (H_0_) was that dental rehabilitation would produce no significant difference in OHRQoL between children with CP and healthy controls, and that no significant change would occur in OHRQoL scores over time within either group.

## 2. Materials and Methods

### 2.1. Study Design and Ethical Approval

This study was designed as a prospective controlled pilot study. The primary purpose of the pilot design was to assess the feasibility of chairside dental rehabilitation and generate preliminary evidence on its effects on OHRQoL in children with CP, in order to inform the design of future large-scale investigations. The study was conducted in accordance with the ethical principles outlined in the Declaration of Helsinki, and the research protocol was approved by the Non-Interventional Clinical Research Ethics Committee of Eskişehir Osmangazi University (ESOGU) (Decision dated 27 February 2024, No. 40). All participating families were informed in detail about the study procedures, potential risks, benefits, and their right to withdraw at any time without any consequences. Written informed consent forms were obtained from the parents or legal guardians of all children participating in the study, and age-appropriate verbal assent was also obtained from children capable of understanding the study procedures. Participant confidentiality and data anonymity were ensured throughout all stages of the study.

### 2.2. Selection and Grouping of Participants

**Figure 1:**
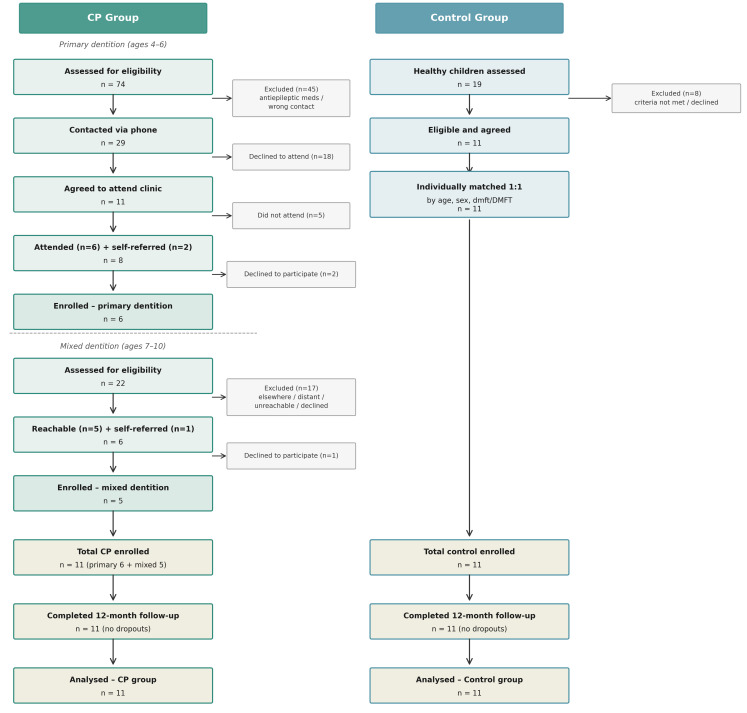
CONSORT flow diagram illustrating the recruitment, screening, and enrollment of participants in the CP and control groups. The CP group was recruited through two separate pathways corresponding to primary (ages 4–6) and mixed dentition (ages 7–10) stages. The control group was individually matched to CP participants based on age, sex, and caries index (dmft/DMFT). All 22 enrolled participants completed the 12-month follow-up period with no dropouts. Solid arrows indicate participant flow through the study stages; the dashed line separates the recruitment pathways for the primary and mixed dentition subgroups within the CP group.

The study was conducted between May 2024 and January 2026 at the Pediatric Neurology clinic of the ESOGU Faculty of Medicine and the Department of Pediatric Dentistry of the Faculty of Dentistry. The study group consisted of children aged 4–10 years who were diagnosed with spastic-type CP, did not use regular medication, possessed functional mobility in at least one upper extremity, and had no additional systemic diseases. The exclusion of children on regular medication was intended to minimize potential confounding, as medications commonly prescribed to children with CP—such as antiepileptics and antispastics—may independently influence oral tissues (e.g., gingival hyperplasia, xerostomia), salivary composition, and oral function, which could interfere with the assessment of OHRQoL outcomes attributable to dental rehabilitation [7,8,12]. The control group was selected from systemically healthy children individually matched with the study group based on age (±6 months), gender, and caries index (dmft/DMFT). A “positive” or “definitely positive” cooperation level (Score 3 or 4) according to the Frankl Behavior Scale was established as a basic criterion for all participants to ensure that treatments could be completed chairside without the need for general anesthesia. The flow of participant recruitment, screening, and enrollment is presented in Figure 1.

### 2.3. Sample Size Calculation

Sample size and power analysis were conducted using G*Power software (version 3.1; Heinrich-Heine-Universität Düsseldorf, Germany). A large effect size (d = 0.80) was assumed based on Cohen’s conventions, which is consistent with the magnitude of differences in OHRQoL scores reported in comparable pediatric populations with special healthcare needs [6]. With a significance level of α = 0.05 and a minimum of 10 participants per group, the statistical power was calculated as 0.81 (81%). Accordingly, 11 participants were included in each group, resulting in a total of 22 participants.

### 2.4. Clinical Evaluation

The neurological status and functional capacities of children with CP were recorded by a single clinical researcher (A.O.E.) pursuing a PhD in the Department of Pediatric Dentistry, using the Gross Motor Function Classification System (GMFCS), the Manual Ability Classification System (MACS), the Communication Function Classification System (CFCS), and the Eating and Drinking Ability Classification System (EDACS) [13,14,15,16].

All intraoral examinations were performed on a standard dental unit in the pediatric dentistry clinic under appropriate overhead lighting, with the aid of a dental mirror and probe. Radiographic examinations (panoramic or periapical radiographs) were performed only when clinically indicated during dental examination and treatment planning.

Through a detailed dental examination, dmft/DMFT scores were determined and documented in the baseline data form. To ensure examiner reliability, a randomly selected subset of participants underwent re-examination after a two-week interval. Intra-examiner agreement was calculated using the kappa statistic, yielding a coefficient of κ = 0.86, indicating strong agreement.

Participants were divided into two subgroups based on their dentition stages: the primary dentition stage (ages 4–6) and the mixed dentition stage (ages 7–10).

### 2.5. Measurement of Oral Health-Related Quality of Life (OHRQoL)

To measure changes in OHRQoL, two different age-specific validated scales were utilized. To prevent interaction between parents and children during the administration of the scales, the forms were completed in separate settings. No suggestive prompts were given to the participants while answering the questions; only standardized explanations were provided as needed.

The 4–6 Age Group: The “Early Childhood Oral Health Impact Scale” (ECOHIS), developed by Pahel et al. [17] and validated into Turkish by Peker et al. [18], was administered. This scale consists of a total of 13 items, where parents evaluate their children’s symptoms, functions, and psychosocial status using a Likert-type scale (0–4). Scores were computed using raw item summation without transformation. Lower total scores indicate better oral health-related quality of life.

The 7–10 Age Group: The “Pediatric Oral Health-Related Quality of Life” (POQL) scale, developed by Huntington et al. [19] and validated into Turkish by Yazıcıoğlu et al. [20], was utilized. This scale consists of child and parent forms that measure physical, social, emotional, and role functioning. Children aged 7 years and older were considered eligible for the POQL; those aged between 7 and 8 years were evaluated on the basis of developmental and cognitive readiness, and administered the POQL in a manner consistent with the 8-year threshold recommended in the original validation study. Scores were computed using raw item summation without transformation. Lower total scores indicate better oral health-related quality of life.

### 2.6. Clinical Procedure and Follow-Up Protocol

All dental treatments and follow-up examinations for the study were performed by A.O.E. to ensure the highest level of standardization and internal consistency among the methods. During the treatment process, the American Academy of Pediatric Dentistry (AAPD) guidelines were taken as a basis; restorative, endodontic, and surgical procedures were completed chairside using standard protocols, while considering the cooperation levels of the children.

Following the completion of treatments, participants were recalled for periodic check-ups at 3, 6, and 12 months. All 22 participants completed the full 12-month follow-up period with no dropouts. In follow-up sessions, the ECOHIS/POQL scales were repeated to determine changes in oral health-related quality of life. No adverse events, unplanned retreatments, or treatment failures were recorded during the 12-month follow-up period.

### 2.7. Statistical Analysis

Statistical analysis of the data was performed using IBM Statistical Package for Social Sciences (SPSS) for Windows, Version 26.0 (IBM Corp., Armonk, NY, USA). The normality of variable distributions was assessed using the Shapiro–Wilk test and visual inspection methods. For normally distributed continuous variables in baseline demographic and clinical comparisons, the independent samples t-test was employed. For OHRQoL scores, non-parametric tests were preferred due to the ordinal nature of the questionnaire data, the small sample size, and the non-normal distribution of scores. The Friedman test was used to evaluate longitudinal changes within the CP and control groups across time points (Baseline, 3, 6, and 12 months). In cases where statistical significance was detected (*p* < 0.05), post hoc analyses with Bonferroni correction were applied to identify the specific measurement intervals responsible for the difference. To complement the *p*-values and support clinical interpretation, effect size (Kendall’s W for Friedman test and r for pairwise comparisons) and 95% confidence intervals were calculated for statistically significant findings. For intergroup comparisons (CP vs. Control), the Mann–Whitney U test was employed for each follow-up period. The level of statistical significance was set at *p* < 0.05 for all analyses.

## 3. Results

A total of 22 children (11 with CP, 11 healthy) were included in the study. In the early childhood group where ECOHIS was administered (6 with CP, 6 healthy), the mean age of the CP group was 4.9 ± 0.8, while that of the control group was 5.3 ± 0.5; this difference was not statistically significant (*p* = 0.250). The gender distribution was 41.7% male and 58.3% female. Upon examination of the baseline clinical data, the dmft scores were 4.5 ± 4.37 in the CP group and 6.66 ± 4.32 in the control group, the difference between them was not significant (*p* = 0.332).

In the school-age group where POQL was administered (5 with CP, 5 healthy), the mean age was 8.5 ± 1.3 in the CP group and 7.8 ± 1.3 in the control group; this difference was not statistically significant (*p* = 0.251). The gender distribution was 20% male and 80% female. Upon examination of baseline clinical data, the mean DMFT and dmft scores were 1.8 ± 2.04 and 4.6 ± 2.5 in the CP group respectively, while they were 2 ± 2 (DMFT) and 5.2 ± 2.86 (dmft) in the control group. No statistically significant difference was detected between these values (DMFT: *p* = 0.911; dmft: *p* = 0.831).

The distribution of dental treatments administered to the participants by group is shown in Table 1. The distribution of dental treatments was similar between the groups.

The functional classification of children with CP across GMFCS, MACS, CFCS, and EDACS levels is presented in Table 2.

The distribution of the participants’ ECOHIS scores measured at baseline and at the 3rd, 6th, and 12th month post-treatment according to the groups is presented in Table 3.

ECOHIS scores remained low across all time points, with no statistically significant differences between the CP and control groups or over time in either the child section or the parent section.

ECOHIS subscale scores showed low median values across all child section domains, with no statistically significant intergroup differences. In the control group, the child function subscale showed an overall change (χ^2^(3) = 9.000, *p* = 0.029, Kendall’s W = 0.500), which did not survive Bonferroni-corrected post hoc analysis. Parent distress and family function subscales remained stable throughout the follow-up. In the CP group, child self-image, social interaction, and family function subdomains were excluded from analysis due to identical ratings across all time points.

The distribution of the participants’ POQL scores measured at baseline and at the 3rd, 6th, and 12th month post-treatment according to the groups is presented in Table 4.

No statistically significant intergroup differences were detected at any time point (Table 4). Parent-reported POQL scores showed statistically significant longitudinal improvements with large effect sizes in both the CP (χ^2^(3) = 13.250, *p* = 0.004, Kendall’s W = 0.883) and control (χ^2^(3) = 12.231, *p* = 0.007, Kendall’s W = 0.815) groups; post hoc analysis identified a significant baseline vs. 12-month difference in both groups (mean rank difference = 2.300, 95% CI [0.701, 3.899], adjusted *p* = 0.029, r = 0.891). Child-reported POQL scores showed no significant change in the CP group (*p* = 0.078), while the control group demonstrated a significant overall change (χ^2^(3) = 9.182, *p* = 0.027, Kendall’s W = 0.612) that did not survive Bonferroni-corrected post hoc analysis.

For the POQL sub-domains, no statistically significant intergroup differences were detected at any time point. Within the role and physical functioning domain, parent-reported scores showed a significant longitudinal change with a large effect size in the CP group (χ^2^(3) = 13.250, *p* = 0.004, Kendall’s W = 0.883), with a significant baseline vs. 12-month improvement (adjusted *p* = 0.029, r = 0.891). In the control group, both child (χ^2^(3) = 8.793, *p* = 0.032, Kendall’s W = 0.586) and parent (χ^2^(3) = 13.909, *p* = 0.003, Kendall’s W = 0.927) scores showed overall changes that did not survive Bonferroni-corrected post hoc analysis. In the social impact and emotional impact sub-domains, scores remained at median 0 throughout the follow-up with no significant change in either group.

## 4. Discussion

The primary objective of this study was to evaluate the impacts of dental rehabilitation on the OHRQoL of children with CP. The literature has demonstrated that OHRQoL levels in children with CP are lower compared to their healthy peers, and this difference is associated with difficulties in maintaining oral hygiene, high caries prevalence, limitations in oral motor functions, and the challenges faced by caregivers [2,7]. However, most existing studies have been limited to cross-sectional designs, and there is a paucity of prospective research monitoring longitudinal changes following dental treatment [9,10]. In this context, the present pilot study aimed to provide preliminary longitudinal evidence on the time-dependent impacts of dental rehabilitation on OHRQoL in children with CP.

The functional classification data obtained here provide important context for interpreting the OHRQoL findings. The predominance of GMFCS Levels 1–3, MACS Levels 1–2, and CFCS/EDACS Level 1 across the CP group confirms that our study sample represented a relatively high-functioning subgroup of children with CP. This profile is consistent with the inclusion criteria, which required functional mobility in at least one upper extremity and adequate cooperation for chairside treatment. As emphasized in the literature, the clinical heterogeneity of CP—particularly differences in gross motor function and manual ability—directly influences oral health outcomes and OHRQoL [3,21]. The lack of a statistically significant baseline difference between the CP and control groups in this study likely reflects these specific characteristics of the sample. The inclusion of only cooperative children with adequate cognitive and motor capacity necessarily excluded participants with more complex clinical presentations, which may have contributed to more favorable baseline scores and a more pronounced perception of post-treatment improvements.

From a biological perspective, the mechanisms underlying the potential improvement in OHRQoL following dental rehabilitation in children with CP may involve several interconnected pathways. The elimination of active carious lesions and the restoration of tooth integrity are expected to reduce orofacial pain and inflammatory burden, thereby directly improving functional capacity and comfort during eating and speaking [12,22]. Furthermore, the reduction in periodontal inflammation through scaling, polishing, and topical fluoride application may contribute to a decrease in systemic inflammatory markers, which has been associated with improved general well-being in pediatric populations [23,24]. In children with CP, where neuromuscular control is already compromised, the alleviation of dental pain may additionally reduce involuntary muscle tension and spasticity-related discomfort, potentially enhancing the child’s participation in daily activities and social interactions [12,22,25]. These biological pathways collectively support the observed trend of improvement in parent-reported OHRQoL scores following comprehensive dental rehabilitation.

The chairside approach adopted in this study stands in contrast to the more commonly reported model of comprehensive dental rehabilitation under general anesthesia (GA) in children with special health care needs. Prior studies have demonstrated that dental rehabilitation under GA is associated with significant improvements in OHRQoL in healthy children [10] and those with special health care needs, with the magnitude of improvement being particularly pronounced in the latter group [9]. However, GA carries inherent anaesthetic risks, resource demands, and logistical barriers that may limit accessibility for many families [26]. The present findings suggest that, for children with high-functioning spastic CP who demonstrate adequate cooperation, chairside rehabilitation may represent a feasible and effective alternative, yielding comparable parent-perceived OHRQoL gains over a 12-month period. Whether similar gains are achievable in lower-functioning CP subgroups who may require GA warrants investigation in future studies.

From a methodological perspective, the prospective controlled design of the present investigation and the use of a control group matched by caries scores stand out as significant strengths. This approach enhances the validity of intergroup comparisons by ensuring the control of a critical confounding factor such as dental caries burden [3]. Furthermore, assessments conducted at four distinct time points—baseline, 3rd, 6th, and 12th months—allow for the monitoring of both the short-term and medium-term effects of dental rehabilitation. Such repeated measures are a robust method for revealing intra-group changes and time-dependent trajectories within each group, which have been rarely implemented in the CP population in the literature [2,27]. When examining the existing evidence in the literature, the study by de Castelo Branco Araújo et al. (2022) remained limited to a matched cross-sectional design, while the recent data from Lansdown et al. (2025) were structured as a broad-scale survey [2,11]. Compared with previous studies, the present pilot study contributes preliminary longitudinal evidence on the outcomes of dental intervention through a 12-month prospective model with repeated follow-up assessments.

The selection of two different OHRQoL instruments—the ECOHIS for the primary dentition group and the POQL for the mixed dentition group [19,28,29]—was necessitated by the developmental differences between the two age groups, as no single instrument is validated across the entire age range covered [30,31]. Rather than combining scores across the two scales, ECOHIS and POQL data were analyzed separately within their respective age subgroups, and no cross-scale conversion or pooling was performed, thereby preserving the developmental validity of each instrument. It should also be noted that alternative or complementary OHRQoL instruments, such as the Child Perceptions Questionnaire for 8–10-year-olds (CPQ8–10) and the Family Impact Scale (FIS), were not employed in this study. The ECOHIS and POQL were selected on the basis of their age-specific validation, availability of Turkish versions, and their established use in pediatric populations with special health care needs [6,18,19,20,32]. The CPQ8–10, originally developed by Jokovic et al., captures child self-reports across four domains-oral symptoms, functional limitations, emotional well-being, and social well-being-and may offer greater sensitivity in school-aged children [31]. The FIS, developed by Locker et al., assesses the impact of children’s oral conditions on family functioning and could complement parent-proxy measures [30]. However, the absence of validated Turkish versions of these instruments at the time of the study precluded their use. Furthermore, the evident floor effects observed in several domains suggest that instruments with greater discriminatory sensitivity in high-functioning pediatric populations warrant consideration in future research.

An additional methodological strength lies in the adoption of a multidimensional measurement model tailored to developmental differences between age groups. In the preschool group (ECOHIS), data were collected via parental perception, whereas the mixed dentition group (POQL) incorporated both children’s self-reports and parental observations. Allowing school-aged children with CP to personally report their own quality of life reinforced the subjective accuracy of the data and provided a more comprehensive perspective than studies based solely on parental reports [7].

The present findings indicate that at baseline, CP group scores were often numerically lower than those of the control group without reaching statistical significance. Parent-reported POQL scores demonstrated a statistically significant improvement in both groups throughout follow-up, while ECOHIS and child-reported POQL scores did not reach statistical significance. These findings warrant discussion in relation to the existing literature, particularly regarding methodological depth and sample homogeneity. For instance, Abanto et al. (2014) reported that oral diseases create distinct negative impacts on the quality of life in children with CP [3]; similarly, El Ashiry et al. (2016) demonstrated through parental perception-based assessments that the CP group had a lower quality of life compared to their healthy peers [7]. Furthermore, in a study conducted by Sruthi et al. (2021) in India, OHRQoL scores of children with CP were found to be significantly higher (indicating poorer quality of life) than those of healthy controls [5]. A vast majority of these studies in the literature do not make a clear distinction between CP types and use broad age ranges, mixing children with clinically diverse degrees of motor impairment under a single group. In contrast, the present study focused exclusively on spastic-type CP and analyzed participants across two distinct developmental subgroups (ages 4–6 and 7–10).

Regarding the ECOHIS results, Du et al. (2010) reported poorer OHRQoL in preschool children with CP compared to healthy controls [6]. In contrast, the present study observed numerically lower (better) ECOHIS scores in the CP group at baseline, although this difference did not reach statistical significance. This discrepancy may be attributed to sample size, cultural differences, and variations in parental prioritization perceptions. In severe neurological conditions such as CP, families often prioritize their child’s general systemic problems and vital function limitations over oral health concerns, which may explain this difference in perception. Caregivers may perceive dental problems as secondary and more tolerable when compared to the complex systemic challenges the child faces in daily life. This situation leads to the reporting of more favorable (lower) quality of life scores that do not fully align with the clinical presentation.

When evaluating the POQL results, Lansdown et al. (2025) reported poorer OHRQoL in children with CP compared to healthy controls in Australia [11]. In the present study, the CP group scored lower at baseline, and improvement was observed in both groups during follow-up, eliminating the between-group difference. This divergence from the broader literature likely reflects the inclusion of a more functional and cooperative CP subgroup in this study.

This study has several limitations. First, the small sample size and the restrictive inclusion criteria—which excluded children with more severe clinical profiles—limit the generalizability of the findings, and the absence of a formal attrition analysis prevents a full assessment of potential selection bias. Second, the absence of an untreated or waitlist control group, together with a statistical approach that did not formally test group-by-time interactions, precludes definitive causal attribution of the observed improvements specifically to dental rehabilitation; mixed-effects modeling in larger future studies would be more appropriate for this purpose. Third, the reliance on parental perception in the scales, together with the lack of blinding of the treating clinician, may have introduced expectancy bias and may not fully capture the children’s subjective experiences. Fourth, floor effects—evidenced by median scores of zero in several domains—may have limited the sensitivity of both instruments in this high-functioning population, and effect size reporting was not extended to all comparisons. Finally, since the study was conducted at a single center, cultural and socioeconomic diversity could not be reflected in the outcomes.

## 5. Conclusions

This 12-month prospective pilot study demonstrates that comprehensive chairside dental rehabilitation is associated with significant improvements in parent-reported OHRQoL in children with spastic-type CP, with outcomes comparable to those observed in healthy peers. While ECOHIS and child-reported POQL scores showed numerical improvements that did not reach statistical significance, the parallel trajectory of improvement across both groups suggests that high-functioning children with CP can benefit from dental rehabilitation in a manner similar to their healthy counterparts. These findings should be interpreted in light of the study’s pilot nature, small sample size, and selective inclusion of high-functioning participants. Future studies with larger and more heterogeneous samples are needed to confirm these preliminary findings and to establish the specific contribution of dental rehabilitation to OHRQoL improvement in children with CP.

## Figures and Tables

**Table 1:** Distribution of dental treatments performed in study and control groups according to the scales used.

	ECOHIS			POQL		
	CP Group (Mean ± SD)	Control Group (Mean ± SD)	*p*-Value	CP Group (Mean ± SD)	Control Group (Mean ± SD)	*p*-Value
**Restorative treatment**	3.6 ± 2.5	6 ± 3.89	0.246	5.6 ± 3.2	5.2 ± 3.03	0.845
**Endodontic treatment**	0.33 ± 0.81	0.5 ± 0.54	0.687	0.4 ± 0.89	0.2 ± 0.44	0.667
**Extraction**	0.5 ± 1.22	0.16 ± 0.4	0.541	0.4 ± 0.54	1.8 ± 2.48	0.254

Data are presented as mean ± standard deviation (SD). The normal distribution assumptions of the baseline demographic and clinical data were verified, and the independent samples *t*-test was employed for inter-group comparisons. The level of statistical significance was set at *p* < 0.05.

**Table 2:** The functional classification of children with CP.

Classification System	Level	n (%)
**GMFCS**	Level 1 (walks without limitations)	4 (36.4)
	Level 2 (walks with limitations)	2 (18.2)
	Level 3 (walks using a hand-held mobility device)	3 (27.3)
	Level 4 (self-mobility with limitations; may use powered mobility)	2 (18.2)
	Level 5 (transported in a manual wheelchair)	0 (0.0)
**MACS**	Level 1 (handles objects easily and successfully)	5 (45.5)
	Level 2 (handles most objects but with somewhat reduced quality and/or speed)	6 (54.5)
	Level 3 (handles objects with difficulty; needs help to prepare and/or modify activities)	0 (0.0)
	Level 4 (handles a limited selection of easily managed objects in adapted situations)	0 (0.0)
	Level 5 (does not handle objects; severely limited ability to perform even simple actions)	0 (0.0)
**CFCS**	Level 1 (effective sender and receiver with unfamiliar and familiar partners)	7 (63.6)
	Level 2 (effective but slower-paced sender and/or receiver with unfamiliar and/or familiar partners)	2 (18.2)
	Level 3 (effective sender and receiver with familiar partners)	2 (18.2)
	Level 4 (inconsistent sender and/or receiver with familiar partners)	0 (0.0)
	Level 5 (seldom effective sender and receiver even with familiar partners)	0 (0.0)
**EDACS**	Level 1 (eats and drinks safely and efficiently)	7 (63.6)
	Level 2 (eats and drinks safely but with some limitations to efficiency)	4 (36.4)
	Level 3 (eats and drinks with some limitations to safety; may have limitations to efficiency)	0 (0.0)
	Level 4 (eats and drinks with significant limitations to safety)	0 (0.0)
	Level 5 (unable to eat and drink safely; tube feeding may be considered)	0 (0.0)

Abbreviations: GMFCS = Gross Motor Function Classification System; MACS = Manual Ability Classification System; CFCS = Communication Function Classification System; EDACS = Eating and Drinking Ability Classification System.

**Table 3:** Distribution of baseline and 3-, 6-, and 12-month post-treatment ECOHIS scores by group.

	CP Group [Median (Min–Max)]	Control Group [Median (Min–Max)]	*p*-Value
**Child Section**			
Baseline	0 (0–5)	2 (0–14)	0.305
3rd month	0 (0–3)	0 (0–1)	0.902
6th month	0 (0–3)	0 (0–1)	0.902
12th month	0 (0–1)	0 (0–2)	0.461
***p *** **-value**	0.145	0.086	
**Parent Section**			
Baseline	0 (0–4)	0.5 (0–11)	0.720
3rd month	0 (0–1)	0 (0–1)	0.523
6th month	0 (0–1)	0 (0–1)	0.523
12th month	0 (0–0)	0 (0–1)	0.138
***p *** **-value**	0.145	0.112	

Mann–Whitney U test was performed for intergroup comparisons (CP vs. Control groups). Friedman test was used to evaluate the longitudinal changes within each group across four time points (Baseline, 3, 6, and 12 months). A *p*-value < 0.05 was considered statistically significant.

**Table 4:** Distribution of baseline and 3-, 6- and 12-month post-treatment POQL scores by group.

	CP Group [Median (Min–Max)]	Control Group [Median (Min–Max)]	*p*-Value
**Child Section**			
Baseline	2.5 (0–2.5)	2.5 (0–25)	0.345
3rd month	0 (0–3.3)	1.7 (0–5)	0.502
6th month	0 (0–0.8)	0 (0–10)	0.699
12th month	0 (0–0.8)	0 (0–3.3)	0.699
***p *** **-value**	0.078	**0.027 †**	
**Parent Section**			
Baseline	1.7 (1.7–6.7) ^a^	5.8 (1.7–20.8) ^a^	0.234
3rd month	0 (0–0.8) ^ab^	0 (0–2.5) ^ab^	0.637
6th month	0 (0–0.8) ^ab^	0 (0–4.2) ^ab^	0.881
12th month	0 (0–0) ^b^	0 (0–0) ^b^	1
***p *** **-value**	0.004 *	0.007 *	

Mann–Whitney U test was performed for intergroup comparisons (CP vs. Control groups). Friedman test was used to evaluate the longitudinal changes within each group across four time points (Baseline, 3, 6, and 12 months). A *p*-value < 0.05 was considered statistically significant. ^a^, ^b^: Time intervals within the same row carrying different letters indicate that the difference between them is statistically significant (*p* < 0.05). † Friedman test result showed an overall significant change (p<0.05); however, Bonferroni-corrected post-hoc pairwise comparisons did not identify statistically significant differences between individual time points. * Friedman test results.

## Data Availability

The data presented in this study are available on request from the corresponding author due to privacy restrictions.
